# The Antitubercular Activities of Natural Products with Fused-Nitrogen-Containing Heterocycles

**DOI:** 10.3390/ph17020211

**Published:** 2024-02-06

**Authors:** Helena I. Boshoff, Neha Malhotra, Clifton E. Barry, Sangmi Oh

**Affiliations:** Tuberculosis Research Section, Laboratory of Clinical Immunology and Microbiology, National Institute of Allergy and Infectious Diseases (NIAID), National Institutes of Health (NIH), Bethesda, MD 20892, USA; hboshoff@niaid.nih.gov (H.I.B.); neha.malhotra@nih.gov (N.M.); cbarry@niaid.nih.gov (C.E.B.III)

**Keywords:** tuberculosis, mycobacteria, natural products, heterocycles, antitubercular activity, antibiotics

## Abstract

Tuberculosis (TB) is notorious as the leading cause of death worldwide due to a single infectious entity and its causative agent, *Mycobacterium tuberculosis* (*Mtb*), has been able to evolve resistance to all existing drugs in the treatment arsenal complicating disease management programs. In drug discovery efforts, natural products are important starting points in generating novel scaffolds that have evolved to specifically bind to vulnerable targets not only in pathogens such as *Mtb*, but also in mammalian targets associated with human diseases. Structural diversity is one of the most attractive features of natural products. This review provides a summary of fused-nitrogen-containing heterocycles found in the natural products reported in the literature that are known to have antitubercular activities. The structurally targeted natural products discussed in this review could provide a revealing insight into novel chemical aspects with novel biological functions for TB drug discovery efforts.

## 1. Introduction 

Tuberculosis (TB) remains a leading cause of death globally due to a single infectious agent with more than 10 million people contracting the disease annually [1]. The COVID-19 pandemic adversely affected surveillance, diagnosis and treatment programs resulting in increased transmission, disease and death due to TB with these numbers slowly returning back to pre-pandemic figures since 2022 [1]. The standard of care for drug-susceptible disease was implemented almost 3 decades ago and consists of a World Health Organization recommended 6-month regimen based on 4 drugs, rifampicin, isoniazid, pyrazinamide and ethambutol which leads to cure in 85% of patients with drug-sensitive disease [1]. Treatment success in patients with drug-resistant TB is much lower and historically consisted of extended duration of treatment (up to 2 years) with drugs associated with significant adverse events. Although the introduction of new antitubercular agents in the last decade has now led to a shorter 6-month regimen of bedaquiline/pretomanid/linezolid/moxifloxacin [1] for the treatment of drug-resistant disease. Unfortunately, resistance to these agents is already emerging and transmission of TB disease with pre-existing bedaquiline resistance has already been observed [2]. The success of novel treatment regimens will be determined by the rate at which resistance will emerge to these drugs. *Mtb* has successfully developed resistance to all clinically available antitubercular drugs in vitro and in vivo [3,4], thus new agents will be needed to combat the inevitable global spread of such resistant strains. 

The development of safe antitubercular regimens that have dramatically shortened treatment durations would conceptually lead to improved treatment adherence, lower economic burdens for patients in public health programs, decreased likelihood of adverse events that arise over extended dosing periods, decreased transmission rates when combined with early diagnosis and decreased rates at which resistance has an opportunity to emerge. In view of this, drugs that inhibit novel targets are less likely to be impacted by pre-existing resistance mechanisms and could potentially target those bacilli in host environments that are recalcitrant to current antitubercular drugs [5]. Antibacterial natural products have evolved in producer organisms to compete with other organisms for nutrients. These metabolites evolved to access and engage essential targets and as such, are usually high affinity ligands for their target protein [6]. Natural products and their derivatives serve as a valuable reservoir of structural diversity enriched with bioactivity and comprise about 75% of clinically available antibacterials [7]. In fact, many drugs and drug candidates of the current TB treatment regimens are based on natural products. For example, the first-line drug, rifampin, is a semisynthetic derivative of the macrolide rifamycins, a polyketide natural product isolated from the soil bacterium *Amycolatopsis rifamycinica* [8]. Other natural products that have been used clinically to treat TB include cycloserine, a broad-spectrum antibiotic isolated from *Streptomyces orchidaceus*, the aminoglycosides produced by different *Streptomyces* species, the β-lactams produced by a variety of fungi and capreomycin produced by *Streptomyces capreolus*. Therefore, continuing efforts to explore natural products in search of structural diversity and mechanistic novelty is an important strategy to expand TB therapeutic options.

Many fused-nitrogen-containing heterocycles like indole, indoline, and tetrahydroisoquinoline are considered to be “privileged structures” because these are molecular scaffolds commonly found in biologically active compounds with versatile interactions with diverse targets [9]. Various fused-nitrogen-containing heterocycles are also important structural features detected in natural products. In this review, we will focus on natural products encompassing fused-nitrogen-containing heterocycles, which were reported to have antitubercular activities against *Mtb* (minimum inhibitory concentration (MIC) < 25 µM with MIC values in this review reflecting growth inhibition of *Mtb* under aerobic actively replicating conditions) without significant cytotoxicity to mammalian cells. 

## 2. Natural Products with Indole Moiety

Despite the wide-ranging structural variety of fused-nitrogen-containing heterocycles, indole unquestionably represents an important privileged structure found in many bioactive natural products and synthetic small-molecules. The global TB drug discovery program is also no exception in terms of actively utilizing indole core for the generation of novel drug candidates with different modes of action including the inhibition of mycobacterial membrane protein large 3 (MmpL3) as well as inhibition of the decaprenylphosphoryl-β-D-ribose 2′-oxidase (DprE1) by a 1,4-azaindole with is currently in clinical trial [10]. In this subsection, not only indole alkaloids but also many cyclopeptides containing tryptophan as a natural indole will be introduced, which have been isolated from natural sources including bacteria, fungi, marine sponges and different plants, and then evaluated their biological activities against mycobacterial species due to wide range of therapeutic potential of indole-containing compounds [11]. 

### 2.1. Tryptophan-Containing Cyclopeptides

Tryptophan is a natural source of indole scaffold that is one of the representative fused-nitrogen-containing heterocycles. Although a group of nucleosidyl-peptide antibiotics, sansanmycins, isolated from *Streptomyces* sp. SS (CGMCC no. 1764) was reported to have moderate antitubercular activities [12,13], this review focuses on the isolation and bioactivity of cyclic peptide natural products containing tryptophan residue due to structural interests.

**Figure 1:**
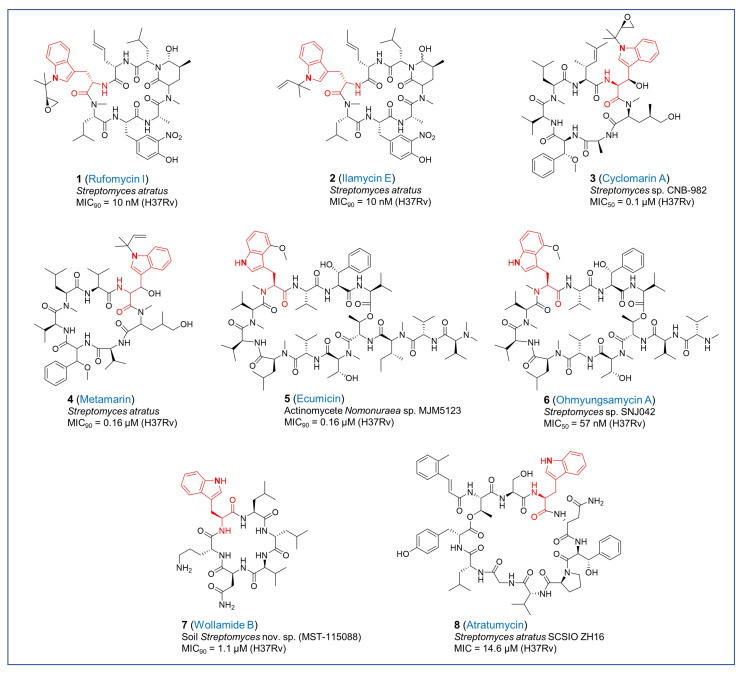
Chemical structures of tryptophan-containing antitubercular cyclopeptides.

The cyclic heptapeptide rufomycins were identified as antimycobacterial natural products produced by *Streptomyces atratus* with some physicochemical characteristics reminiscent of the ilamycins. Rufomycins are selective for *Mtb* exerting no inhibition of other Gram-positive or Gram-negative bacteria, fungi, yeast or mammalian cells [14,15]. Rufomycins and ilamycins were later shown to be synonymous for a series of 40 actinomycete-produced cyclic heptapeptides containing an isoprenyl-modified tryptophan which is often further oxidized to an epoxide (as in rufomycin I in Figure 1) or a diol as well as in most analogs, a 3-nitrotyrosine where the most potent antimycobacterial analogs have a hydroxy-methylamidopiperidinone formed by cyclization of the side chain of leucine 5 with the nitrogen of leucine 6 [16,17]. The MIC_90_ of ilamycin E (**2**, Figure 1) against *Mtb* is 10 nM [18] where MIC_90_ is the concentration that results in ≥90% growth inhibition. The MIC_90_ of rufomycin I (**1**, Figure 1) against *Mtb* is 10~20 nM [16,19,20], it is similarly potent against a range of mycobacteria [16] and, of note, is active against both *Mtb* and *M. abscessus* during growth in host macrophages [16], highlighting the ability of the compound to cross not only the mycobacterial cell wall and cell membrane but also eukaryotic membranes. Despite modestly better pharmacokinetic (PK) properties compared to ecumicin, the efficacy of rufomycins against *Mtb* in vivo was not reported [16]. A study in *M. abscessus*-infected mice revealed a somewhat lower bacterial burden in rufomycin I treated mice compared to untreated controls with some of the effects attributed to an undefined host-mediated effect of these peptides as evidenced by lower inflammation levels and higher expression of autophagy/lysosomal genes [21].

The rufomycins, like the cyclomarins and ecumicin, disrupt cellular metabolism by targeting the ClpC1 component of the ClpC1:ClpP1P2 proteasome-like supercomplex. The ClpC1 component acts as the gateway to the proteolytic ClpP1P2 barrel by recognizing proteins targeted for degradation and unfolding them by an adenosine triphosphate (ATP)-mediated mechanism [22]. The binding sites of ecumicin and the rufomycins are both in the *N*-terminal domain of ClpC1 with the binding of either natural product affecting the binding of the other. However, rufomycin-resistant mutants bearing ClpC1 mutations do not confer cross-resistance to ecumicin. The X-ray structure of the *N*-terminal domain of ClpC1 bound to rufomycin showed that the binding site largely overlaps with the cyclomarin binding site although their modes of binding differ [19]. For rufomycin I, the epoxide opens up to form a covalent bond to the sulfur of the first methionine of the protein [19], the importance of which remains unclear since the epoxy group does not appear to be essential for the antitubercular activity of rufomycin analogs [17,20]. Despite the similarity in binding site to cyclomarin, rufomycins do not activate the ATPase activity of ClpC1 as observed with cyclomarin. The binding of the rufomycins to the essential hexameric ClpC1 AAA+ ATPase interferes with its binding to ClpP1P2 thereby decreasing the proteolytic activity of the ClpP1P2 proteolytic component [16,23]. Surprisingly, in contrast to other ClpP1-targeting natural products such as ecumicin and cyclomarin, the rufomycins have poor activity against non-replicating *Mtb* despite their cidal effect on replicating *Mtb* [16,17] demonstrating that the mode of inhibition of proteostasis through ClpC1 and ClpP1P2 dysregulation can have different cellular outcomes.

Cyclomarin is a non-ribosomal cyclic heptapeptide produced by *Streptomyces* sp. CNB-982 [24] and also produced by other bacteria such as *Salinispora arenicola* [25]. Cyclomarin A (**3**, Figure 1) has a MIC_50_ of 0.1 µM against *Mtb* and is also cidal against hypoxic non-replicating *Mtb* (90% kill in 5 days at 2.5 µM) [26]. Attempts to generate resistant mutants indicated that resistance does not arise readily underscoring the essentiality of its target [26]. This antibiotic is not cytotoxic and is specific for mycobacteria since other Gram-positive and Gram-negative bacteria tested were not susceptible. Similar to the rufomycins, the cyclomarins contain an *N*-prenylated tryptophan which can be oxidized to the corresponding epoxy moiety [25]. Cyclomarin targets the essential ClpC1 ATPase resulting in activation of its ATPase activity by binding to the loop linking the two α-helical domains of the *N*-terminal domain of ClpC1 [27]. The hydrophobic binding pocket of cyclomarin A (**3**) is specific for ClpC1 and is not found in Gram-positive ClpC orthologs explaining the mycobacterial selectivity of this cyclic peptide [27]. Cyclomarin binding to ClpC1 stimulates proteolysis by ClpP1P2 [28,29]. The X-ray structure of cyclomarin bound to the *N*-terminal domain of ClpC1 [27] and comparison with the rufomycin-ClpC1 crystal structure indicated that both these cyclic peptides formed a covalent bond with the sulfur of methionine 1 through their epoxide moieties although the binding interactions with the ClpC1 amino acids at the binding site were different [19]. Despite this, the epoxide in cyclomarin is not essential for bioactivity [26,30,31] possibly because its binding to ClpC1, irrespective of the covalent bond, is stronger than that of rufomycin [19]. Metamarin (**4**, Figure 1), produced by the same soil actinomycete that produces the rufomycins and ilamycins, is a cyclomarin analog where the epoxide on the tryptophan is replaced with a double bond and has a MIC of 0.16 µM against *Mtb* [32]. A cryo-electron microscopy (cryo-EM) study of a stabilized ClpC1 hexamer bound to cyclomarin showed that the binding of this natural product did not affect protein conformation generating a structure indistinguishable from the apo protein complex [33]. A parallel study revealed that cyclomarin induces ClpC1 transition from an inactive non-hexameric state into higher-order assembled hexameric rings that activate ClpP1P2 proteolytic activity [29,34]. The known cell permeability of cyclomarin combined with the structural information on ClpC1 binding and the residues that are critical for this interaction, enabled the design of bacterial proteolysis targeting chimeras (BacPROTACs) consisting of a cyclomarin-protein-of-interest-ligand chimera which targeted intracellular mycobacterial proteins of interest for proteolytic digestion [31]. Proteomic analysis of the consequences of growth inhibition with cyclomarin indicated extensive protein degradation in mycobacterial cells although compensatory upregulation of ClpC2 and ClpC3, proteins with homology to the *N*-terminal ClpC1 domain was observed [34]. These proteins protect cells from dysregulated proteolysis under conditions of stress associated with increased levels of misfolded proteins and the cyclomarin binding site in ClpC2 protects cells from this antibiotic by reducing effective cytosolic concentrations. The conserved cyclomarin binding site of ClpC2 and ClpC1 allowed the design of BacPROTACs consisting of two cyclomarin analogs fused by a linker. These dimeric homo-BacPROTACs led to simultaneous degradation of both ClpC1 and ClpC2 were more potent than cyclomarin itself against *Mtb* (MIC_50_ = 0.3 µM) and were equipotent in a non-replicating model of *Mtb* induced by ATP depletion [34].

Ecumicin (**5**, Figure 1) is a non-ribosomal tridecapeptide produced by actinomycete *Nomonuraea* sp. MJM5123. This depsipeptide potently inhibits the growth of mycobacteria with MIC_90_ values in the 0.13~0.6 µM range against *Mtb* (MIC_90_ for *Mtb* H37Rv = 0.16 µM) [35]. The MIC value was only minimally decreased in the presence of protein [35]. Ecumicin (**5**) was found to have no activity against other Gram-positive and Gram-negative bacteria as well as yeast. This natural product kills both replicating and non-replicating *Mtb* as well as *Mtb* growing in macrophages with an almost 2-log kill observed in infected cells over 7 days at the MIC value [35]. Ecumicin (**5**) is not cytotoxic and is stable in human and mouse microsomes [35] but, in common with most peptide-based natural products, has poor predicted oral bioavailability. Thus, in vivo efficacy testing required subcutaneous administration of a polymeric micellar formulation, which resulted in accumulation in the lung after consecutive dosing. Nevertheless, despite the remarkable efficacy against *Mtb* growing in macrophages, intermittent dosing over 12 doses in acutely infected mice was unable to fully inhibit bacterial replication in mice although bacterial lung burdens were more than a log lower than the vehicle control group at 20 mg/kg. 

*Nomonuraea* sp. MJM5123 was shown to produce several analogs of ecumicin all of which had similar MIC values against *Mtb* with similar binding affinities to ClpC1 [19,36]. The limited structure-activity relationship (SAR) evident from these studies suggested that the hydroxylation of phenylalanine to yield *threo*-β-hydroxy-L-phenylalanine was not essential for bioactivity since deoxyecumicin retained equipotent *Mtb* activity. In fact, substitution of *N*-methyl-L-phenylalanine led to a more than 2-fold increase in potency [37]. In addition, the 4-methoxy group on tryptophan was not important for potency against *Mtb* [37]. Substitution of the third position *N*-methyl-L-*allo*-isoleucine with *N*-methyl valine or *N*-methyl-L-leucine did not affect potency [36,37]. The eight-position *N*-methyl-L-valine could be substituted with various groups allowing for simpler synthetic routes to this natural product where aromatic or aliphatic sidechains were tolerated although removal, replacement with amino acids containing an alcohol, acid, amine or with constrained side chains decreased potency [37]. In fact, the replacement of the eight-position *N*-methyl-L-valine with alanine led to a compound that was superior to ecumicin (**5**) in stimulating ClpC1 ATPase activity as well as in its in vitro cidality. This analog showed some efficacy in a zebrafish model of *M. marinum* infection although a direct comparison to the parent compound ecumicin (**5**) was lacking [37].

Resistance to ecumicin does not arise readily and resistant mutants were only obtained after exposure of cells to stepwise increases in drug concentration. The resistant mutants were found to have mutations in the *N*-terminal domain of ClpC1 (L92S, L92F or L96P). Further evidence for the essential ClpC1 ATPase being the target was the ability of ecumicin to protect this protein in lysates from pronase digestion as well as its potent stimulation of the ATPase activity of ClpC1 while inhibiting proteolysis by ClpP1P2 similar to the mode of inhibition observed with the cyclic peptide lassomycin [23,38]. A cryo-EM study of a stabilized ClpC1 hexamer bound to ecumicin suggested that binding resulted in a structural reorganization of the *N*-terminal domains similar to that predicted to occur during substrate binding [33]. Despite the apparent differences in the mode of ClpC1 binding, ecumicin also results in the formation of higher-order hexameric ClpC1 complexes [34]. Although activation of the ClpC1 ATPase activity appears to be the mechanism of growth inhibition, Hoi and coworkers revealed that inhibition of proteolysis occurred with some substrates although other substrates were readily degraded [34]. 

Ohmyungsamycin A (**6**, Figure 1), produced by another actinomycete, *Streptomyces* sp. SNJ042 is structurally similar to ecumicin where the terminal *N*,*N*-dimethyl valine is replaced with *N*-methyl valine as well as lacking the third position *N*-methyl-L-*allo*-isoleucine. The analog ohmyungsamycin B contains an *N*,*N*-dimethyl valine instead of *N*-methyl valine and has similar potency against *Mtb* (MIC_50_ = 33~100 nM for A and B derivatives) [39]. The valine-valine side is not essential for activity since its removal results in only a 6-fold loss of antitubercular potency [39]. Surprisingly, this cyclic depsipeptide, in addition to its potent antimycobacterial activity (*Mtb* MIC_50_ = 57 nM), also inhibits the growth of several eukaryotic cancer cell lines, which suggested that this natural product could be optimized as a cancer drug [40,41]. Activity against mammalian cells through an unknown target affecting cell cycle arrest [42] is; however, unlikely to be a desirable property for an antibacterial compound. The eukaryotic activity further contributes to innate antimicrobial responses by stimulating autophagy in infected macrophages [41] and stimulating inflammatory responses in *M. abscessus*-infected macrophages [43] reminiscent of the host-directed effects reported for rufomycin [21]. Similar to ecumicin, ohmyungsamycin A (**6**) has in vivo efficacy as shown by enhanced host survival during the treatment of *M. marinum*-infected flies [41]. The SAR analyses completed on ecumicin (**5**) to facilitate total synthesis provide valuable information on its antitubercular SAR [37]. The mechanism of action in mycobacteria is assumed to be similar to that of ecumicin although the additional effects on mammalian cells could suggest additional off-target effects.

The soil actinomycete *Streptomyces* nov. sp. (MST-115088) was found to produce six related cyclic hexapeptides namely desotamide, desotamides B, E and F as well as wollamides A and B (**7**, Figure 1). All these cyclic hexapeptides were found to have no apparent cytotoxicity to eukaryotic cells while inhibiting the growth of certain Gram-positive bacteria although only the positively charged ornithine-containing wollamides inhibited the growth of *M. bovis* BCG with MIC_50_ values of 2.8 and 3.1 µM for wollamide A and B (**7**), respectively [44]. Wollamides were found to be cidal against *M. bovis* BCG in vitro and wollamide B (**7**) had a very modest growth inhibitory effect against *M. bovis* BCG growing in macrophages [44]. A subsequent analysis of wollamide B (**7**) indicated a MIC_90_ of 1.1 µM against *Mtb,* with modestly favorable PK and ADMET properties, prompting a further SAR evaluation of this cyclic peptide. The evaluation revealed that all amino acids played a role in bioactivity although certain replacements could be tolerated while maintaining favorable ADMET properties [45]. A follow-up study confirmed the similar potency of wollamide A against in vitro growing as well as intramacrophage *Mtb* but two wollamide analogs which were synthesized to improve solubility and PK properties had no effect in an acute mouse model of *Mtb* infection [46]. Further studies to explore the SAR of the wollamides underscored the importance of the positive charge of the 6th position where replacement of the ornithine with D-lysine or D-arginine was tolerated although the L-stereoisomers ablated activity. Only the D-arginine replacement retained activity against intramacrophage *Mtb* [47]. This study also revealed the cidal activity of these peptides on non-replicating *Mtb*. Currently, the target of the wollamides remains unclear. The biggest hurdle facing wollamide optimization is the development of an analog with improved PK properties. 

Atratumycin (**8**, Figure 1) was discovered in extracts from *Streptomyces atratus* SCSIO ZH16 [48]. This is a cyclic depsipeptide containing an unusual 2-alkenyl cinnamoyl modification inhibiting *Mtb* growth with a MIC of 3.8~14.6 µM against *Mtb* H37Ra and H37Rv, respectively. No inhibition was observed against other Gram-positive or Gram-negative bacteria as well as a variety of eukaryotic cell lines suggesting either a *Mtb*-specific target or binding site or selective uptake by *Mtb*. Changes in the length of the cinnamoyl modification sowed some effects on MIC with an increase in 4 carbon units giving an almost 7-fold increase in potency [49], which could reflect improved target engagement or better cellular penetration.

### 2.2. Other Indole Derivatives

**Figure 2:**
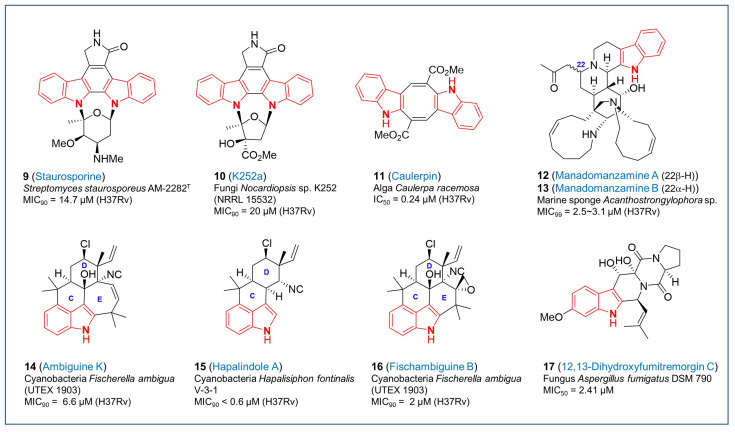
Chemical structures of antitubercular natural products with indole moiety.

Indole-containing secondary metabolites, indole alkaloids, are one of the important subclasses consisting of diverse natural products. Due to structural interests based on validated therapeutic outcomes of indole moiety, they have been evaluated on their biological activities in different disease models including *Mycobacteria*. Bisindole alkaloids are structurally complex dimers comprised of two indole units, which can be indirectly connected to various structures or result from direct polymerization of monomeric units. These are more complex structures as compared to their corresponding monomeric units and the bisindole is produced at a late stage in the biosynthetic process. These alkaloids exhibit diverse pharmacological properties including anticancer, antimalarial, antileishmanial, antibacterial and antimycobacterial activities [50,51,52,53,54,55,56,57]. Staurosporine (**9**, Figure 2) having the indolocarbazole core was originally isolated from *Streptomyces staurosporeus* AM-2282^T^ obtained from soil samples collected in Japan in 1977 [58]. Biosynthetically, the indolocarbazole aglycone moiety of staurosporines is derived from two molecules of tryptophan while the sugar component is derived from glucose and methionine [59]. Staurosporine (**9**) and its related natural analog K252a (**10**, Figure 2), isolated from culture broths of *Nocardiopsis* sp. K252 was identified to possess broad-spectrum inhibitory activities against protein kinases [60,61,62,63]. K252a (**10**) represents a similar indolocarbazole structure as staurosporine with a difference in the side chains of the sugar moiety. The inhibitory activities of staurosporine (**9**), K252a (**10**) and their derivatives are attributed to their strong binding with the ATP binding pockets of kinases in their active conformation [64,65,66]. Due to their potent inhibitory activities against protein kinases, researchers studied their influence on the growth of *Mtb* as it contains a high number of eukaryotic-type serine/threonine kinases, two-component systems and tyrosine kinases. A study by Guo et al. in 2009 demonstrated a modest activity of staurosporine (**9**) against *Mtb* H37Rv with a MIC_90_ value of 14.7 μM [67]. Likewise, K252a (**10**) also exhibited modest antitubercular activity against *Mtb* H37Rv (MIC_90_ = 20 μM) [68]. It seems likely that staurosporine and K252a (**10**) being potent kinase inhibitors might have toxic effects on human cells, which is supported by their cytotoxic effects in many cancer cell lines as reported in the literature. Nevertheless, these alkaloids have not been reported to inhibit the growth of normal human cell lines. Further work to develop highly specific staurosporine derivatives against *Mtb* would mitigate concerns about off-target effects and would enable the inclusion of inhibitors in regimens that engage targets predicted to be essential for in vivo pathogenesis [69]. 

Caulerpin (**11**, Figure 2), another bisindole alkaloid, is an orange-red pigment first isolated from the green alga *Caulerpa racemose* and the red alga *Chondria armat*, which features two indole moieties fused to a central cyclooctatetraene diester moiety [70]. Caulerpin (**11**) has two methyl groups esterifying an α,β-unsaturated system and was identified as dimethyl-7,14-dihydrodibenzo-[α,β]-phenazine-6,13-dicarboxylate. It has demonstrated no acute toxicity in Swiss mice [71] and RAW 246.7 macrophages [72]. The antitubercular activity of caulerpin and its synthetic analogs was first demonstrated by Canché Chay et al. in 2014 by showing growth inhibition of *Mtb* H37Rv [73]. Caulerpin (**11**) had an excellent IC_50_ value of 0.24 µM better than the control drug rifampin (IC_50_ = 0.55 µM). Notably, caulerpin (**11**) demonstrated about 80% inhibition of *Mtb* H37Rv cells without serious cytotoxicity, indicating its potential as a lead molecule for antitubercular drug development. Nevertheless, caulerpins have been reported to affect a diversity of biological processes in both prokaryotes and eukaryotes with no defined targets demonstrated to date, which is a considerable challenge for efforts in developing highly selective antitubercular compounds.

Manadomanzamine A (**12**, Figure 2) and B (**13**, Figure 2) are heterocyclic β-carboline alkaloids derived from a novel structural rearrangement of the manzamine scaffold. Both have the same molecular formula but differ in the stereochemistry at position C22. They were first discovered from the Indonesian marine sponges *Acanthostrongylophora* sp. and the microbes associated with them [74]. These are representatives of a novel class of manzamines possessing strong activity against *Mtb*, (MIC_99_ = 3.1 µM for A and 2.5 µM for B). In addition, they also exhibited potent activity against HIV and AIDS opportunistic fungal infections like *Candida albicans* and *Cryptococcus neoformans*. Although they showed anticancer effects on several cancer cell lines, both manadomanzamines A (**12**) and B (**13**) did not exhibit cytotoxicity against Vero cells [74]. Owing to their strong anti-*Mtb* potential along with their inhibitory activity against HIV- and AIDS-associated fungi, manadomanzamines A (**12**) and B (**13**) are potent lead molecules for tuberculosis drug development. Chemical synthesis to obtain these alkaloids has so far not been successful and has only been isolated from the sponges or the microbes associated with them. 

Indole alkaloids isolated from cyanobacteria are another family of biologically relevant natural products classified into hapalindoles, fischerindoles, ambiguines and welwitindolinones. They are all comprised of tetra or pentacyclic scaffolds derived from tryptophan and geranyl pyrophosphate, and contain an indole or oxindole core, a highly functionalized cyclohexane unit fused to the indole at C3, isonitrile or isothiocyanate groups and varying levels of oxidation. Of these polycyclic compounds, a few members of the hapalindoles and ambiguines isonitrile alkaloids have been shown to possess antitubercular activity with tolerable cytotoxicity and therefore, could be interesting lead molecules for developing antitubercular drugs. Mo et al. demonstrated in their study that ambiguine K (**14**, Figure 2) an isonitrile isolated from culture extracts of the cyanobacterium *Fischerella ambigua* (UTEX 1903) exhibited antitubercular activity against *Mtb* H37Rv (MIC_90_ = 6.6 μM) with tolerable cytotoxicity against Vero cells (IC_50_ = 53.2 μM) [75]. Structurally, ambiguines are a class of hapalindole-type natural products that feature a 1,1-dimethylallyl unit bound to C2 of the indole moiety. The isoprene moiety cyclizes to form an additional seven-membered ring. It is worth mentioning here that apart from ambiguine K (**14**), two other members of this class demonstrated comparable anti-*Mtb* activity and minimal cytotoxicity including ambiguine C and M (MIC_90_ = 7.0 and 7.5 μM, respectively) [75]. Ambiguine C is a tetracyclic alkaloid without the E ring and ambiguine M is a hydroxylated analog of ambiguine K, at the double bond of the E ring. Hapalindole A (**15**, Figure 2) is the prototype of this family of compounds as it was the first of its kind to be isolated from *Hapalisiphon fontinalis* V-3-1 bearing antialgal and antimycotic activities [76]. Culture extracts from cyanobacteria *Westiellopsis* sp. (SAG 20.93) and *Fischeralla musicola* (UTEX LB1829) were later demonstrated to possess excellent activity against *Mtb* H37Rv with a MIC_90_ value less than 0.6 µM, whereas no cytotoxicity was associated with it [77]. Other molecules in the series that showed potent antitubercular activity without any serious cytotoxicity against Vero cells included hapalindole I (MIC_90_ = 2.0 µM) with double bond in the D ring, hapalindole G (MIC_90_ = 6.8 µM) as a diastereomer of hapalindole A, and hapalindole X (MIC_90_ = 2.5 µM) without chloride but with different vinyl and aryl substituents in D ring [75,77]. Fischambiguine B (**16**, Figure 2), another hapalindole-type alkaloid and structurally related to ambiguine isonitriles, was first obtained from cultured *Fischerella ambigua* (UTEX 1903). It has an isoprene unit at the C2 of the indole moiety that might undergo further cyclization to form other ambiguines. Interestingly, this newly identified alkaloid displayed potent inhibitory activity against *Mtb* with a MIC_90_ of 2 μM, while no inhibition of other microbes and no cytotoxicity was detected in Vero cells. Structural analogy with other members in the group present in the literature, which did not exhibit any antitubercular activity emphasized the significance of spiro-epoxy group in the six-membered ring E of fischambiguine B (**16**) to be most likely responsible for its *Mtb* killing activity [78]. Elucidation of the mechanism of action of these hapalindoles would motivate further development.

In the category of indole alkaloids, 12,13-dihydroxyfumitremorgin C (**17**, Figure 2) is another potent antitubercular lead, first obtained from the resting cultures of fungus *Aspergillus fumigatus* DSM 790 [79]. It is a heteropentacyclic compound derived from fumitremorgin C, which is substituted by two hydroxyl groups at positions 12 and 13. Feeding experiments identified tryptophan, proline, and mevalonic acid as the starting material involving specific cytochrome oxidases for 12,13-dihydroxyfumitremorgin C (**17**) biosynthesis [80]. Cultures from deep sea-derived *Aspergillus* sp. SCSIO Ind09F01 resulted in the production of 12,13-dihydroxyfumitremorgin C (**17**) that exhibited antitubercular activity with a MIC_50_ of 2.41 μM although its mechanism of action remains unknown [81].

## 3. Natural Products with Other Fused-Nitrogen-Containing Heterocycles

**Figure 3:**
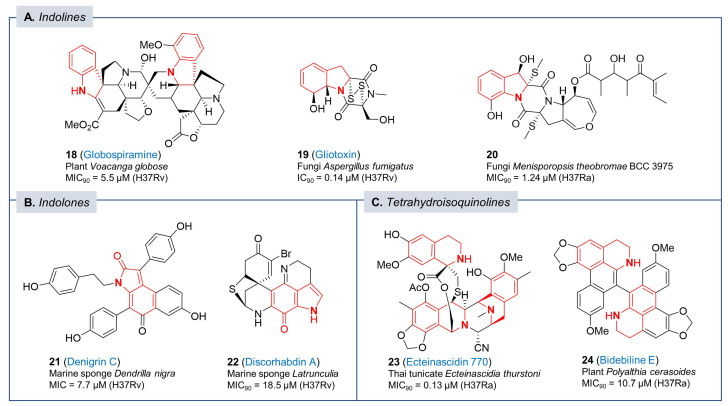
Chemical structures of antitubercular natural products with (**A**,**B**) oxidized or reduced indoles and (**C**) tetrahydroisoquinoline moiety.

Like the indole moiety, oxidized or reduced indoles like indoline and various indolones are commonly found in bioactive alkaloid natural products. Globospiramine (**18**, Figure 3) is a spirobisindole alkaloid having an *Aspidosperma−Aspidosperma* skeleton with indolines that was isolated from a Philippine plant, *Voacanga globose*. It has antitubercular activity with a MIC_90_ of 5.5 µM against *Mtb* H37Rv as well as activity against non-replicating bacteria based on the low-oxygen recovery assay with a MIC of 7.1 µM [82]. Gliotoxin (**19**, Figure 3) is a sulfur-containing mycotoxin that has a fused scaffold of diketopiperazine and an indoline. Various pathogenic fungi, including *Aspergillus fumigatus*, are known to produce this bridged disulfide metabolite [83]. Although gliotoxin (**19**) shows general toxicity to mammalian cells (IC_90_ = 8.2 µM), it was confirmed to have promising activity with an IC_90_ of 0.14 µM against *Mtb* expressing GFP compared to other bacterial species. Gliotoxin likely has multiple targets in *Mtb* although it is a non-competitive inhibitor of the mycothiol-S-conjugate amidase [84]. The disulfide bond in the structure may be required for specific antitubercular activity because a methylated analog lacks the disulfide lost activity, indicating that the disulfide bond is essential for its antitubercular activity [85]. Another indoline derivative, **20** (Figure 3), has a structural pattern similar to that of gliotoxin including an indoline-fused 1,4-cyclohexadione with sulfur-containing substituents. It was one of the metabolites isolated from the seed fungus *Menisporopsis theobromae* BCC 3975 and the structure was elucidated by in-depth spectroscopic data. Not only antimalarial activity, but also antimycobacterial activity of compound **20** was evaluated and confirmed to show potent MIC_90_ with 1.24 µM without serious cytotoxicity against human cancer cell lines [86]. Nevertheless, the development of gliotoxins as a drug remains a formidable challenge based on the known off-target effects of these redox-active compounds.

Denigrin C (**21**, Figure 3) was a new compound isolated from the ethyl acetate extract of the marine sponge *Dendrilla nigra* by bioactivity-guided screening. Among the isolated 3,4-diaryl pyrrole alkaloids, this indolone-type compound proved to have good potency against *Mtb* H37Rv with a MIC value of 7.7 µM [87]. Another indolone derivative, discorhabdin A (**22**, Figure 3) was a pyrroloiminoquinone alkaloid isolated from a sponge species of the genus *Latrunculia*. Structurally, discorhabdin A has a unique sulfur-containing fused ring system incorporating the azacarbocyclic spirocyclohexadienone and pyrroloiminoquinone system with a bromine substituent. Due to its potent biological activities, synthetic efforts toward the discorhabdin alkaloids were reported along with their biological evaluation [88]. The antitubercular activity of discorhabdin A (**22**) was reported to be broad spectrum with antimicrobial activities against MRSA, *M. intracellulare*, and *Mtb* (H37Rv, MIC_90_ = 18.5 µM) [89]. 

Ecteinascidin 770 (**23**, Figure 3) was isolated from the potassium cyanide-pretreated Thai tunicate *Ecteinascidia thurstoni* and its structure was fully elucidated by extensive 2D NMR analysis. It contains three tetrahydroisoquinoline subunits with potent antitubercular activity against *Mtb* H37Ra at MIC_90_ of 0.13 μM [90]. Notably, the ecteinascidin family consists of many biologically active compounds and specifically, ecteinascidin 743, an analog having a hydroxyl instead of the nitril group in ecteinascidin 770 (**23**). Ecteinascidin 743 was approved as an antitumor drug for the treatment of advanced soft-tissue sarcoma and ovarian cancer under the brand name Yondelis. Currently, ecteinascidin 743 is commercially manufactured by a semi-synthetic process from cyanosafracin B based on the pioneering synthetic studies reported by the Corey laboratory [91]. Mechanism of action studies would inform on the potential to develop ecteinascidin analogs with high *Mtb* selectivity with no predicted mammalian off-target effects.

In 2007, Kanokmedhakul et al. isolated bidebiline E (**24**, Figure 3), a new dimeric aporphine alkaloid, from the roots of the evergreen tree *Polyalthia cerasoides*. After structural elucidation on the basis of NMR and mass spectroscopy, they evaluated the antimycobacterial activity against *Mtb* H37Ra with a MIC_90_ of 10.7 μM but, cytotoxicity against human cells was not performed to establish selectivity for further evaluation [92]. 

**Figure 4:**
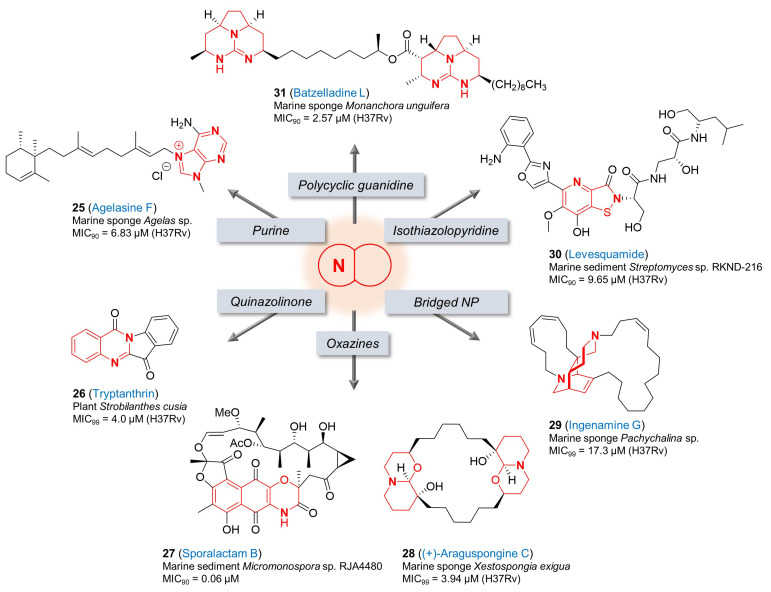
Chemical structures of antitubercular natural products with various fused-nitrogen-containing heterocycles.

An orange *Agelas* sp. sponge is a natural source containing agelasine F (**25**, Figure 4), a monocyclic diterpenoid with a 9-methyladenium core. The MICs of this purine analog were evaluated against drug-susceptible *Mtb* (MIC_90_ = 6.8 µM) and isolates resistant to individual first-line treatments (rifampin, isoniazid, pyrazinamide, and ethambutol) (MIC_90_ = 6.8~13.6 µM). Its antitubercular activity was confirmed against *Mtb*-infected macrophages with an EC_90_ value of 28.6 µM that was below the level of cytotoxicity to Vero cells (IC_50_ = 74.2 µM) [93], although further improvements on this selectivity index would be critical for progression in the drug discovery pipeline. Due to its biological potential, synthetic and medicinal chemistry efforts around the core of agelasines have been published to improve its antitubercular activities [94,95]. 

Tryptanthrin (**26**, Figure 4) was isolated from the Chinese herb *Strobilanthes cusia*, and has been studied as a promising hit for the development of novel TB drugs because of its high bactericidal activity on both drug-susceptible and drug-resistant *Mtb* strains, simple structure and ease of synthesis. This natural alkaloid possesses a quinazolinone scaffold and is active against *Mtb* H37Rv with a MIC_99_ of 4.0 μM and MBC of 4 μM. Substantial medicinal chemistry efforts to improve the antitubercular activity have been performed and a few analogs with improved activities were developed [96,97]. However, several potential issues including solubility, PK and toxicity remain to be improved to enable in vivo efficacy determination. The cellular target(s) of tryptanthrin (**26**) are not known. In 2012, enoyl-acyl carrier protein reductase (InhA) was suggested as the target of tryptanthrins by in silico modeling [98]. In another study by Frolova et al., spontaneous resistant mutants of a tryptanthrin analog in *M. smegmatis* were identified in which mutations in *MSMEG_1963* (EmbR transcriptional regulator) led to high-level resistance, while those in *MSMEG_5597* (TetR transcriptional regulator) led to low-level resistance. In this study, it was also established that the MmpS5-MmpL5 efflux system was able to provide resistance to tryptanthrin (**26**) and its analogs [99] although the mechanism of action and identification of potential molecular targets remains unknown.

The isolation and antitubercular activity of sporalactam B (**27**, Figure 4) was introduced by Williams et al. in 2017 [100]. From the ethyl acetate extracts of solid agar cultures of a *Micromonospora* sp. RJA4480 isolated from marine sediment, several new natural ansa (having a looped handle or chain) macrolide antibiotics including sporalactams A and B (**27**) having a tricyclic benzoxazine core were purified. Sporalactam B (**27**) exhibited promising antibiotic activity against not only MRSA and *E. coli* but also *Mtb* (MIC_90_ = 0.06 µM). Its antitubercular activity was also confirmed against the pathogen in infected macrophages with a MIC of 3~9 µM without any cytotoxicity to the cells [100]. This finding suggests that sporalactam B (**27**) could represent a promising candidate for the development of new antibiotics including for the treatment of TB. 

(+)-Araguspongine C (**28**, Figure 4) is one of the bis-1-oxaquinolizidine compounds in the family of araguspongine/xestospongin alkaloids [101]. It was isolated from the marine sponge *Xestospongia exigua*, and shown to inhibit *Mtb* H37Rv with a MIC_99_ of 3.94 μM [102]. Interestingly, araguspongine C (**28**) is also known to possess diverse important biological activities, for example, inhibition of rat brain nitric oxide synthase activity *in vitro*, vasodilation activity, inhibition of the inositol 1,4,5- trisphosphate receptor and the endoplasmic-reticulum Ca^2+^ pumps [103,104], and antiproliferative effects in breast cancer cells through suppression of c-Met and HER2 receptor tyrosine kinase signaling [105]. The diversity of potential targets is a concern for the development of highly selective antitubercular compounds based on this scaffold.

Ingenamine G (**29**, Figure 4) was a new nitrogenous metabolite reported by de Oliveira et al. after the chemical investigation of biologically active methanol extract of the marine sponge *Pachychalina* sp., and its antitubercular activity was reported against *Mtb* H37Rv (MIC_99_ = 17.3 µM) in addition to its anticancer effects on several cancer cell lines [106]. According to the Baldwin–Whitehead hypothesis, the biosynthesis of many polycyclic marine alkaloids like ingenamine G (**29**), manadomanzamine A (**12**) and B (**13**) having etheno-bridged diazadecaline core is speculated to derive from partly reduced macrocyclic alkylpyridine intermediates via a transannular Diels–Alder reaction [107]. Structural interests and their biological activities led to synthetic efforts to develop ingenamine derivatives [108,109]. The potency against both cancer cell lines and *Mtb* is not an advantage for antitubercular drug discovery.

The structure of levesquamide (**30**, Figure 4) contains a rare isothiazolinone moiety fused with pyridine. This polyketide non-ribosomal peptide (PK-NRP) hybrid marine natural product was isolated from the marine sediment *Streptomyces* sp. RKND-216 and its structural elucidation were performed through NMR spectroscopy supported by an analysis of the biosynthetic gene cluster (BGC) and stable-isotope labeling experiments [110]. Liang et al. reported that levesquamide (**30**) exhibited antimicrobial activity against rifampicin- and isoniazid-resistant strains (MIC_90_ = 9.46 and 9.90 μM, respectively) as well as drug-sensitive *Mtb* H37Rv (MIC_90_ = 9.65 μM). In addition, it retained activity in the low oxygen recovery assay (LORA) with a MIC of 22.28 μM without cytotoxicity to human cell lines [110]. Due to structural interests and biological activity, Jiang et al. recently reported the total synthesis of levesquamide (**30**) for the first time [111]. 

After the isolation of five new polycyclic guanidine alkaloids from the Caribbean sponge *Monanchora unguifera* by Hua et al., their biological activities were confirmed against cancer, protozoa, HIV-1 and AIDS-associated opportunistic infectious pathogens, including *Mtb*. Among them, batzelladine L (**31**, Figure 4) showed the most potent activity with a MIC_90_ of 2.57 µM in the antituberculosis assay against *Mtb*. Its antimicrobial activity was also tested against *M. intracellulare* and a MIC_90_ of 0.47 µM was determined [112]. Synthetic efforts towards these polycyclic guanidine alkaloids and structurally related congeners have been reported to support their various in vitro biological interests including antimicrobial activities [113]. As above, the wide range of biological activities ascribed to this scaffold is a concern in the development of an *Mtb*-selective lead.

## 4. Conclusions

Natural products with nitrogen-containing heterocycles have a proven record for excellent antitubercular activity with mechanistic novelty. Because of their structural complexity such starting points for drug development for TB are rarely pursued seriously. In general, the size of these molecules seems to be associated with lower frequencies of emergence of resistance than purely synthetic drugs offering one more incentive to motivate lead optimization in this space. Even if these are only considered as a target selection activity they are still of incredibly high value since the mechanisms selected for mycobacterial killing in nature are likely to be the points of highest vulnerability. This would be especially true for natural products that have a high selectivity index for bacteria compared to mammalian cells. Targeted collections from environmental niches in which slow-growing mycobacteria occur naturally are likely to reveal novel chemistry and highly evolved new leads for future drug development efforts. Several natural products have activity against both replicating as well as non-replicating bacilli with a notable example from this review being the cyclomarins. These compounds would be predicted to have activity against *Mtb* residing in the different lesions that define the spectrum of human disease. Environmental niches where growth is further restricted by poor nutrient availability or adverse exogenous stresses such as low pH could potentially harbor microorganisms that secrete natural products with activity against metabolic processes important for non-replicating persistence. One could speculate that such compounds could lead to therapies that target the slowly replicating or non-replicating bacilli that are thought to characterize latent disease.

The formidable challenges of progressing a natural product from discovery to clinical development have skewed drug discovery efforts to favor synthetic candidates. This is evident from the clinical pipeline for TB drugs [114] where two out of five, zero out of seven and one out of twelve new drugs in the pre-clinical, phase I and phase II stages, respectively, are natural product-derived. Of these three, one (sanfetrinem) is a repurposed β-lactam whereas the spectinamides are semi-synthetic derivatives of the spectinomycin antibiotic. The development of spectinomycins into derivatives with antitubercular activity highlights the success of structure-guided drug discovery combined with capitalizing on knowledge of mycobacterial metabolism, in this case, efflux [115]. Progression of natural products based on nitrogen-containing heterocycles into the clinical pipeline would benefit from a structure-guided design already exemplified by some of the cyclomarin derivatives discussed, understanding of the influx/efflux/xenobiotic transformation mechanisms as well as efforts to improve ADMET properties without compromising on-target and selectivity properties.

## Data Availability

Not applicable.

## References

[1] World Health Organization (2023). The WHO Global Tuberculosis Report 2023.

[2] Derendinger B., Dippenaar A., de Vos M., Huo S., Alberts R., Tadokera R., Limberis J., Sirgel F., Dolby T., Spies C. (2023). Bedaquiline resistance in patients with drug-resistant tuberculosis in Cape Town, South Africa: A retrospective longitudinal cohort study. Lancet Microbe.

[3] Gygli S.M., Borrell S., Trauner A., Gagneux S. (2017). Antimicrobial resistance in *Mycobacterium tuberculosis*: Mechanistic and evolutionary perspectives. FEMS Microbiol. Rev..

[4] Mancuso G., Midiri A., De Gaetano S., Ponzo E., Biondo C. (2023). Tackling Drug-Resistant Tuberculosis: New Challenges from the Old Pathogen *Mycobacterium tuberculosis*. Microorganisms.

[5] Walesch S., Birkelbach J., Jézéquel G., Haeckl F.P.J., Hegemann J.D., Hesterkamp T., Hirsch A.K.H., Hammann P., Müller R. (2023). Fighting antibiotic resistance-strategies and (pre)clinical developments to find new antibacterials. EMBO Rep..

[6] Walsh C.T. (2018). Nature Builds Macrocycles and Heterocycles into Its Antimicrobial Frameworks: Deciphering Biosynthetic Strategy. ACS Infect. Dis..

[7] Atanasov A.G., Zotchev S.B., Dirsch V.M., Supuran C.T., the International Natural Product Sciences Taskforce (2021). Natural products in drug discovery: Advances and opportunities. Nat. Rev. Drug Discov..

[8] Surette M.D., Spanogiannopoulos P., Wright G.D. (2021). The Enzymes of the Rifamycin Antibiotic Resistome. Accout. Chem. Res..

[9] Skoreński M., Sieńczyk M. (2021). The Fellowship of Privileged Scaffolds—One Structure to Inhibit Them All. Pharmaceuticals.

[10] Bajad N.G., Singh S.K., Singh S.K., Singh T.D., Singh M. (2022). Indole: A promising scaffold for the discovery and development of potential anti-tubercular agents. Curr. Res. Pharmacol. Drug Discov..

[11] Umer S.M., Solangi M., Khan K.M., Saleem R.S.Z. (2022). Indole-Containing Natural Products 2019-2022: Isolations, Reappraisals, Syntheses, and Biological Activities. Molecules.

[12] Xie Y., Xu H., Sun C., Yu Y., Chen R. (2010). Two novel nucleosidyl-peptide antibiotics: Sansanmycin F and G produced by *Streptomyces* sp SS. J. Antibiot..

[13] Tran A.T., Watson E.E., Pujari V., Conroy T., Dowman L.J., Giltrap A.M., Pang A., Wong W.R., Linington R.G., Mahapatra S. (2017). Sansanmycin natural product analogues as potent and selective anti-mycobacterials that inhibit lipid I biosynthesis. Nat. Commun..

[14] Shibata M., Yamamoto H., Higashidani E., Nakazawa K. (1962). Studies on Streptomycetes: Part I. *Streptomyces atratus* Nov. Sp., Producing New Antituberculous Antibiotics Rufomycin A and B. Agric. Biol. Chem..

[15] Higashidani E., Ueyanagi J., Shibata M., Nakazawa K., Miyake A., Iwasaki H., Yamamoto H. (1962). Studies on Streptomycetes: Part II. Rufomycin A and B, New Antituberculous Antibiotics. Agric. Biol. Chem..

[16] Choules M.P., Wolf N.M., Lee H., Anderson J.R., Grzelak E.M., Wang Y., Ma R., Gao W., McAlpine J.B., Jin Y.-Y. (2019). Rufomycin Targets ClpC1 Proteolysis in *Mycobacterium tuberculosis* and *M. abscessus*. Antimicrob. Agents Chemother..

[17] Zhou B., Achanta P.S., Shetye G., Chen S.-N., Lee H., Jin Y.-Y., Cheng J., Lee M.-J., Suh J.-W., Cho S. (2021). Rufomycins or Ilamycins: Naming Clarifications and Definitive Structural Assignments. J. Nat. Prod..

[18] Ma J., Huang H., Xie Y., Liu Z., Zhao J., Zhang C., Jia Y., Zhang Y., Zhang H., Zhang T. (2017). Biosynthesis of ilamycins featuring unusual building blocks and engineered production of enhanced anti-tuberculosis agents. Nat. Commun..

[19] Wolf N.M., Lee H., Choules M.P., Pauli G.F., Phansalkar R., Anderson J.R., Gao W., Ren J., Santarsiero B.D., Lee H. (2019). High-Resolution Structure of ClpC1-Rufomycin and Ligand Binding Studies Provide a Framework to Design and Optimize Anti-Tuberculosis Leads. ACS Infect. Dis..

[20] Zhou B., Shetye G., Yu Y., Santarsiero B.D., Klein L.L., Abad-Zapatero C., Wolf N.M., Cheng J., Jin Y., Lee H. (2020). Antimycobacterial Rufomycin Analogues from *Streptomyces atratus* Strain MJM3502. J. Nat. Prod..

[21] Park C.R., Paik S., Kim Y.J., Kim J.K., Jeon S.M., Lee S.-H., Whang J., Cheng J., Suh J.-W., Cao J. (2021). Rufomycin Exhibits Dual Effects Against *Mycobacterium abscessus* Infection by Inducing Host Defense and Antimicrobial Activities. Front. Microbiol..

[22] Lupoli T.J., Vaubourgeix J., Burns-Huang K., Gold B. (2018). Targeting the Proteostasis Network for Mycobacterial Drug Discovery. ACS Infect. Dis..

[23] Hong J., Duc N.M., Jeong B.-C., Cho S., Shetye G., Cao J., Lee H., Jeong C., Lee H., Suh J.-W. (2023). Identification of the inhibitory mechanism of ecumicin and rufomycin 4-7 on the proteolytic activity of *Mycobacterium tuberculosis* ClpC1/ClpP1/ClpP2 complex. Tuberculosis.

[24] Renner M.K., Shen Y.C., Cheng X.C., Jensen P.R., Frankmoelle W., Kauffman C.A., Fenical W., Lobkovsky E., Clardy J. (1999). Cyclomarins A–C, new antiinflammatory cyclic peptides produced by a marine bacterium (*Streptomyces* sp.). J. Am. Chem. Soc..

[25] Schultz A.W., Oh D.-C., Carney J.R., Williamson R.T., Udwary D.W., Jensen P.R., Gould S.J., Fenical W., Moore B.S. (2008). Biosynthesis and Structures of Cyclomarins and Cyclomarazines, Prenylated Cyclic Peptides of Marine Actinobacterial Origin. J. Am. Chem. Soc..

[26] Schmitt E.K., Riwanto M., Sambandamurthy V., Roggo S., Miault C., Zwingelstein C., Krastel P., Noble C., Beer D., Rao S.P. (2011). The natural product cyclomarin kills *Mycobacterium tuberculosis* by targeting the ClpC1 subunit of the caseinolytic protease. Angew. Chem. Int. Ed. Engl..

[27] Vasudevan D., Rao S.P., Noble C.G. (2013). Structural Basis of Mycobacterial Inhibition by Cyclomarin A. J Biol. Chem..

[28] Maurer M., Linder D., Franke K.B., Jager J., Taylor G., Gloge F., Gremer S., Le Breton L., Mayer M.P., Weber-Ban E. (2019). Toxic Activation of an AAA+ Protease by the Antibacterial Drug Cyclomarin A. Cell Chem. Biol..

[29] Taylor G., Frommherz Y., Katikaridis P., Layer D., Sinning I., Carroni M., Weber-Ban E., Mogk A. (2022). Antibacterial peptide CyclomarinA creates toxicity by deregulating the *Mycobacterium tuberculosis* ClpC1-ClpP1P2 protease. J. Biol. Chem..

[30] Barbie P., Kazmaier U. (2016). Total Synthesis of Cyclomarin A, a Marine Cycloheptapeptide with Anti-Tuberculosis and Anti-Malaria Activity. Org. Lett..

[31] Morreale F.E., Kleine S., Leodolter J., Junker S., Hoi D.M., Ovchinnikov S., Okun A., Kley J., Kurzbauer R., Junk L. (2022). BacPROTACs mediate targeted protein degradation in bacteria. Cell.

[32] Li L., MacIntyre L.W., Ali T., Russo R., Koirala B., Hernandez Y., Brady S.F. (2021). Biosynthetic Interrogation of Soil Metagenomes Reveals Metamarin, an Uncommon Cyclomarin Congener with Activity against *Mycobacterium tuberculosis*. J. Nat. Prod..

[33] Weinhaupl K., Gragera M., Bueno-Carrasco M.T., Arranz R., Krandor O., Akopian T., Soares R., Rubin E., Felix J., Fraga H. (2022). Structure of the drug target ClpC1 unfoldase in action provides insights on antibiotic mechanism of action. J. Biol. Chem..

[34] Hoi D.M., Junker S., Junk L., Schwechel K., Fischel K., Podlesainski D., Hawkins P.M.E., van Geelen L., Kaschani F., Leodolter J. (2023). Clp-targeting BacPROTACs impair mycobacterial proteostasis and survival. Cell.

[35] Gao W., Kim J.-Y., Anderson J.R., Akopian T., Hong S., Jin Y.-Y., Kandror O., Kim J.-W., Lee I.-A., Lee S.-Y. (2015). The Cyclic Peptide Ecumicin Targeting ClpC1 Is Active against *Mycobacterium tuberculosis* In Vivo. Antimicrob. Agents Chemother..

[36] Gao W., McAlpine J.B., Choules M.P., Napolitano J.G., Lankin D.C., Simmler C., Ho N.A., Lee H., Suh J.-W., Burton I.W. (2017). Structural Sequencing of Oligopeptides Aided by ^1^H Iterative Full-Spin Analysis. J. Nat. Prod..

[37] Hawkins P.M.E., Hoi D.M., Cheung C.Y., Wang T., Quan D., Sasi V.M., Liu D.Y., Linington R.G., Jackson C.J., Oehlers S.H. (2022). Potent Bactericidal Antimycobacterials Targeting the Chaperone ClpC1 Based on the Depsipeptide Natural Products Ecumicin and Ohmyungsamycin A. J. Med. Chem..

[38] Gavrish E., Sit C.S., Cao S., Kandror O., Spoering A., Peoples A., Ling L., Fetterman A., Hughes D., Bissell A. (2014). Lassomycin, a Ribosomally Synthesized Cyclic Peptide, Kills *Mycobacterium tuberculosis* by Targeting the ATP-Dependent Protease ClpC1P1P2. Chem. Biol..

[39] Khalil Z.G., Salim A.A., Lacey E., Blumenthal A., Capon R.J. (2014). Wollamides: Antimycobacterial Cyclic Hexapeptides from an Australian Soil *Streptomyces*. Org. Lett..

[40] Asfaw H., Laqua K., Walkowska A.M., Cunningham F., Martinez-Martinez M.S., Cuevas-Zurita J.C., Ballell-Pages L., Imming P. (2017). Design, synthesis and structure-activity relationship study of wollamide B; a new potential anti TB agent. PLoS ONE.

[41] Asfaw H., Wetzlar T., Martinez-Martinez M.S., Imming P. (2018). An efficient synthetic route for preparation of antimycobacterial wollamides and evaluation of their in vitro and in vivo efficacy. Bioorg. Med. Chem. Lett..

[42] Khalil Z.G., Hill T.A., De Leon Rodriguez L.M., Lohman R.J., Hoang H.N., Reiling N., Hillemann D., Brimble M.A., Fairlie D.P., Blumenthal A. (2019). Structure-Activity Relationships of Wollamide Cyclic Hexapeptides with Activity against Drug-Resistant and Intracellular *Mycobacterium tuberculosis*. Antimicrob. Agents Chemother..

[43] Hur J., Jang J., Sim J., Son W.S., Ahn H.-C., Kim T.S., Shin Y.-H., Lim C., Lee S., An H. (2018). Conformation-Enabled Total Syntheses of Ohmyungsamycins A and B and Structural Revision of Ohmyungsamycin B. Angew. Chem. Int. Ed. Engl..

[44] Um S., Choi T.J., Kim H., Kim B.Y., Kim S.-H., Lee S.K., Oh K.-B., Shin J., Oh D.-C. (2013). Ohmyungsamycins A and B: Cytotoxic and antimicrobial cyclic peptides produced by *Streptomyces* sp. from a volcanic island. J. Org. Chem..

[45] Kim T.S., Shin Y.-H., Lee H.-M., Kim J.K., Choe J.H., Jang J.-C., Um S., Jin H.S., Komatsu M., Cha G.-H. (2017). Ohmyungsamycins promote antimicrobial responses through autophagy activation via AMP-activated protein kinase pathway. Sci. Rep..

[46] Byun W.S., Kim S., Shin Y.-H., Kim W.K., Oh D.-C., Lee S.K. (2020). Antitumor Activity of Ohmyungsamycin A through the Regulation of the Skp2-p27 Axis and MCM4 in Human Colorectal Cancer Cells. J. Nat. Prod..

[47] Jeon S.M., Kim Y.J., Nguyen T.Q., Cui J., Thi Bich Hanh B., Silwal P., Kim J.K., Kim J.M., Oh D.-C., Jang J. (2022). Ohmyungsamycin promotes M1-like inflammatory responses to enhance host defense against *Mycobacteroides abscessus* infections. Virulence.

[48] Sun C., Yang Z., Zhang C., Liu Z., He J., Liu Q., Zhang T., Ju J., Ma J. (2019). Genome Mining of *Streptomyces atratus* SCSIO ZH16: Discovery of Atratumycin and Identification of Its Biosynthetic Gene Cluster. Org. Lett..

[49] Yang Z., Sun C., Liu Z., Liu Q., Zhang T., Ju J., Ma J. (2020). Production of Antitubercular Depsipeptides via Biosynthetic Engineering of Cinnamoyl Units. J. Nat. Prod..

[50] Muñoz V., Moretti C., Sauvain M., Caron C., Porzel A., Massiot G., Richard B., Le Men-Olivier L. (1994). Isolation of bis-indole alkaloids with antileishmanial and antibacterial activities from *Peschiera van heurkii* (syn. *Tabernaemontana van heurkii*). Planta Med..

[51] Oh K.-B., Mar W., Kim S., Kim J.-Y., Lee T.-H., Kim J.-G., Shin D., Sim C.J., Shin J. (2006). Antimicrobial Activity and Cytotoxicity of Bis(indole) Alkaloids from the Sponge *Spongosorites* sp. Biol. Pharm. Bull..

[52] Fernandez L.S., Buchanan M.S., Carroll A.R., Feng Y.J., Quinn R.J., Avery V.M. (2009). Flinderoles A–C: Antimalarial Bis-indole Alkaloids from *Flindersia* Species. Org. Lett..

[53] Zhang Y., Hu C. (2020). Anticancer activity of bisindole alkaloids derived from natural sources and synthetic bisindole hybrids. Arch. Pharm..

[54] Ramkissoon A., Seepersaud M., Maxwell A., Jayaraman J., Ramsubhag A. (2020). Isolation and Antibacterial Activity of Indole Alkaloids from *Pseudomonas aeruginosa* UWI-1. Molecules.

[55] Xu M., Peng R., Min Q., Hui S., Chen X., Yang G., Qin S. (2022). Bisindole natural products: A vital source for the development of new anticancer drugs. Eur. J. Med. Chem..

[56] Khan N.A., Kaur N., Owens P., Thomas O.P., Boyd A. (2022). Bis-Indole Alkaloids Isolated from the Sponge *Spongosorites calcicola* Disrupt Cell Membranes of MRSA. Int. J. Mol. Sci..

[57] Guzmán-Gutiérrez S.L., Silva-Miranda M., Krengel F., Huerta-Salazar E., León-Santiago M., Díaz-Cantón J.K., Espitia Pinzón C., Reyes-Chilpa R. (2022). Antimycobacterial Activity of Alkaloids and Extracts from *Tabernaemontana alba* and *T. arborea*. Planta Med..

[58] Omura S., Iwai Y., Hirano A., Nakagawa A., Awaya J., Tsuchya H., Takahashi Y., Masuma R. (1977). A new alkaloid AM-2282 of *Streptomyces* origin. Taxonomy, fermentation, isolation and preliminary characterization. J. Antibiot..

[59] Nakano H., Omura S. (2009). Chemical biology of natural indolocarbazole products: 30 years since the discovery of staurosporine. J. Antibiot..

[60] Tamaoki T., Nomoto H., Takahashi I., Kato Y., Morimoto M., Tomita F. (1986). Staurosporine, a potent inhibitor of phospholipid/Ca++dependent protein kinase. Biochem. Biophys. Res. Commun..

[61] Kase H., Iwahashi K., Matsuda Y. (1986). K-252a, a potent inhibitor of protein kinase C from microbial origin. J. Antibiot..

[62] Tapley P., Lamballe F., Barbacid M. (1992). K252a is a selective inhibitor of the tyrosine protein kinase activity of the trk family of oncogenes and neurotrophin receptors. Oncogene.

[63] Lawrie A.M., Noble M.E., Tunnah P., Brown N.R., Johnson L.N., Endicott J.A. (1997). Protein kinase inhibition by staurosporine revealed in details of the molecular interaction with CDK2. Nat. Struct. Biol..

[64] Zhao B., Bower M.J., McDevitt P.J., Zhao H., Davis S.T., Johanson K.O., Green S.M., Concha N.O., Zhou B.B. (2002). Structural basis for Chk1 inhibition by UCN-01. J. Biol. Chem..

[65] Atwell S., Adams J.M., Badger J., Buchanan M.D., Feil I.K., Froning K.J., Gao X., Hendle J., Keegan K., Leon B.C. (2004). A Novel Mode of Gleevec Binding Is Revealed by the Structure of Spleen Tyrosine Kinase. J. Biol. Chem..

[66] Tanramluk D., Schreyer A., Pitt W.R., Blundell T.L. (2009). On the Origins of Enzyme Inhibitor Selectivity and Promiscuity: A Case Study of Protein Kinase Binding to Staurosporine. Chem. Biol. Drug Des..

[67] Guo S., Tipparaju S.K., Pegan S.D., Wan B., Mo S., Orjala J., Mesecar A.D., Franzblau S.G., Kozikowski A.P. (2009). Natural Product Leads for Drug Discovery: Isolation, Synthesis and Biological Evaluation of 6-Cyano-5-Methoxyindolo[2,3-*a*]carbazole Based Ligands as Antibacterial Agents. Bioorg. Med. Chem..

[68] Fernandez P., Saint-Joanis B., Barilone N., Jackson M., Gicquel B., Cole S.T., Alzari P.M. (2006). The Ser/Thr protein kinase PknB is essential for sustaining mycobacterial growth. J. Bacteriol..

[69] Mori M., Sammartino J.C., Costantino L., Gelain A., Meneghetti F., Villa S., Chiarelli L.R. (2019). An Overview on the Potential Antimycobacterial Agents Targeting Serine/Threonine Protein Kinases from *Mycobacterium tuberculosis*. Curr. Top. Med. Chem..

[70] Aguilar-Santos G. (1970). Caulerpin, a new red pigment from green algae of the genus *Caulerpa*. J. Chem. Soc. Perkin Trans..

[71] Vidal J.P., Laurent D., Kabore S.A., Rechencq E., Boucard M., Girard J.P., Escale R., Rossi J.C. (1984). Caulerpin, Caulerpicin, *Caulerpa scalpelliformis*: Comparative Acute Toxicity Study. Bot. Mar..

[72] Nagappan T., Vairappan C.S. (2014). Nutritional and bioactive properties of three edible species of green algae, genus *Caulerpa* (Caulerpaceae). J. Appl Phycol..

[73] Canché Chay C.I., Gómez Cansino R., Espitia Pinzón C.I., Torres-Ochoa R.O., Martínez R. (2014). Synthesis and Anti-Tuberculosis Activity of the Marine Natural Product Caulerpin and Its Analogues. Mar. Drugs.

[74] Peng J., Hu J.-F., Kazi A.B., Li Z., Avery M., Peraud O., Hill R.T., Franzblau S.G., Zhang F., Schinazi R.F. (2003). Manadomanzamines A and B: A Novel Alkaloid Ring System with Potent Activity against Mycobacteria and HIV-1. J. Am. Chem. Soc..

[75] Mo S., Krunic A., Chlipala G., Orjala J. (2009). Antimicrobial ambiguine isonitriles from the cyanobacterium *Fischerella ambigua*. J. Nat. Prod..

[76] Moore R.E., Cheuk C., Patterson G.M.L. (1984). Hapalindoles: New Alkaloids from the Blue-Green-Alga *Hapalosiphon Fontinalis*. J. Am. Chem. Soc..

[77] Kim H., Lantvit D., Hwang C.H., Kroll D.J., Swanson S.M., Franzblau S.G., Orjala J. (2012). Indole alkaloids from two cultured cyanobacteria, *Westiellopsis* sp. and *Fischerella muscicola*. Bioorg. Med. Chem..

[78] Mo S., Krunic A., Santarsiero B.D., Franzblau S.G., Orjala J. (2010). Hapalindole-related alkaloids from the cultured cyanobacterium *Fischerella ambigua*. Phytochemistry.

[79] Abraham W.-R., Arfmann H.A. (1990). 12,13-Dihydroxy-fumitremorgin-C from *Aspergillus-fumigatus*. Phytochemistry.

[80] Li S.-M. (2011). Genome mining and biosynthesis of fumitremorgin-type alkaloids in ascomycetes. J. Antibiot..

[81] Luo X., Zhou X., Lin X., Qin X., Zhang T., Wang J., Tu Z., Yang B., Liao S., Tian Y. (2017). Antituberculosis compounds from a deep-sea-derived fungus *Aspergillus* sp. SCSIO Ind09F01. Nat. Prod. Res..

[82] Macabeo A.P., Vidar W.S., Chen X., Decker M., Heilmann J., Wan B., Franzblau S.G., Galvez E.V., Aguinaldo M.A., Cordell G.A. (2011). *Mycobacterium tuberculosis* and cholinesterase inhibitors from *Voacanga globosa*. Eur. J. Med. Chem..

[83] Kwon-Chung K.J., Sugui J.A. (2009). What do we know about the role of gliotoxin in the pathobiology of *Aspergillus fumigatus*?. Med. Mycol..

[84] Fu J., Luo X., Lin M., Xiao Z., Huang L., Wang J., Zhu Y., Liu Y., Huaming Tao H. (2023). Marine-Fungi-Derived Gliotoxin Promotes Autophagy to Suppress *Mycobacteria tuberculosis* Infection in Macrophage. Mar. Drugs.

[85] Stanley S.A., Grant S.S., Kawate T., Iwase N., Shimizu M., Wivagg C., Silvis M., Kazyanskaya E., Aquadro J., Golas A. (2012). Identification of novel inhibitors of *M. tuberculosis* growth using whole cell based high-throughput screening. ACS Chem. Biol..

[86] Chinworrungsee M., Kittakoop P., Saenboonrueng J., Kongsaeree P., Thebtaranonth Y. (2006). Bioactive compounds from the seed fungus *Menisporopsis theobromae* BCC 3975. J. Nat. Prod..

[87] Murali Krishna Kumar M., Devilal Naik J., Satyavathi K., Ramana H., Raghuveer Varma P., Purna Nagasree K., Smitha D., Venkata Rao D. (2014). Denigrins A–C: New antitubercular 3,4-diarylpyrrole alkaloids from *Dendrilla nigra*. Nat. Prod. Res..

[88] Wada Y., Fujioka H., Kita Y. (2010). Synthesis of the Marine Pyrroloiminoquinone Alkaloids, Discorhabdins. Mar. Drugs.

[89] Na M., Ding Y., Wang B., Tekwani B.L., Schinazi R.F., Franzblau S., Kelly M., Stone R., Li X.-C., Ferreira D. (2010). Anti-infective discorhabdins from a deep-water alaskan sponge of the genus *Latrunculia*. J. Nat. Prod..

[90] Suwanborirux K., Charupant K., Amnuoypol S., Pummangura S., Kubo A., Saito N. (2002). Ecteinascidins 770 and 786 from the Thai tunicate *Ecteinascidia thurstoni*. J. Nat. Prod..

[91] Le V.H., Inai M., Williams R.M., Kan T. (2015). Ecteinascidins. A Review of the Chemistry, Biology and Clinical Utility of Potent Tetrahydroisoquinoline Antitumor Antibiotics. Nat. Prod. Rep..

[92] Kanokmedhakul S., Kanokmedhakul K., Lekphrom R. (2007). Bioactive Constituents of the Roots of *Polyalthia cerasoides*. J. Nat. Prod..

[93] Mangalindan G.C., Talaue M.T., Cruz L.J., Franzblau S.G., Adams L.B., Richardson A.D., Ireland C.M., Concepcion G.P. (2000). Agelasine F from a Philippine *Agelas* sp. Sponge Exhibits in vitro Antituberculosis Activity. Planta Med..

[94] Bakkestuen A.K., Gundersen L.L., Petersen D., Utenova B.T., Vik A. (2005). Synthesis and antimycobacterial activity of agelasine E and analogs. Org. Biomol. Chem..

[95] Vik A., Hedner E., Charnock C., Samuelsen O., Larsson R., Gundersen L.L., Bohlin L. (2006). (+)-Agelasine D: Improved Synthesis and Evaluation of Antibacterial and Cytotoxic Activities. J. Nat. Prod..

[96] Duca G., Pogrebnoi S., Boldescu V., Aksakal F., Uncu A., Valica V., Uncu L., Negres S., Nicolescu F., Macaev F. (2019). Tryptanthrin Analogues as Inhibitors of Enoyl-acyl Carrier Protein Reductase: Activity against *Mycobacterium tuberculosis*, Toxicity, Modeling of Enzyme Binding. Curr. Top. Med. Chem..

[97] Hwang J.-M., Oh T., Kaneko T., Upton A.M., Franzblau S.G., Ma Z., Cho S.-N., Kim P. (2013). Design, Synthesis, and Structure–Activity Relationship Studies of Tryptanthrins as Antitubercular Agents. J. Nat. Prod..

[98] Tripathi A., Wadia N., Bindal D., Jana T. (2012). Docking studies on novel alkaloid tryptanthrin and its analogues against enoyl-acyl carrier protein reductase (InhA) of *Mycobacterium tuberculosis*. Indian J. Biochem. Biophys..

[99] Frolova S.G., Klimina K.M., Kumar R., Vatlin A.A., Salunke D.B., Kendrekar P., Danilenko V.N., Maslov D.A. (2020). Identification of Mutations Conferring Tryptanthrin Resistance to *Mycobacterium smegmatis*. Antibiotics.

[100] Williams D.E., Dalisay D.S., Chen J., Polishchuck E.A., Patrick B.O., Narula G., Ko M., Av-Gay Y., Li H., Magarvey N. (2017). Aminorifamycins and Sporalactams Produced in Culture by a *Micromonospora* sp. Isolated from a Northeastern-Pacific Marine Sediment Are Potent Antibiotics. Org. Lett..

[101] Althagbi H.I., Alarif W.M., Al-Footy K.O., Abdel-Lateff A. (2020). Marine-Derived Macrocyclic Alkaloids (MDMAs): Chemical and Biological Diversity. Mar. Drugs.

[102] Orabi K.Y., El Sayed K.A., Hamann M.T., Dunbar D.C., Al-Said M.S., Higa T., Kelly M. (2002). Araguspongines K and L, new bioactive bis-1-oxaquinolizidine *N*-oxide alkaloids from Red Sea specimens of *Xestospongia exigua*. J. Nat. Prod..

[103] Ismatullah H., Jabeen I., Muhammad Tariq Saeed M.T. (2021). Biological Regulatory Network (BRN) Analysis and Molecular Docking Simulations to Probe the Modulation of IP_3_R Mediated Ca^2+^ Signaling in Cancer. Genes.

[104] Wang Z.-J., Zhao F., Wang C.-F., Zhang X.-M., Xiao Y., Zhou F., Wu M.-N., Zhang J., Qi J.-S., Yang W. (2019). Xestospongin C, a Reversible IP3 Receptor Antagonist, Alleviates the Cognitive and Pathological Impairments in APP/PS1 Mice of Alzheimer’s Disease. J. Alzheimers Dis..

[105] Akl M.R., Ayoub N.M., Ebrahim H.Y., Mohyeldin M.M., Orabi K.Y., Foudah A.I., El Sayed K.A. (2015). Araguspongine C induces autophagic death in breast cancer cells through suppression of c-Met and HER2 receptor tyrosine kinase signaling. Mar. Drugs.

[106] de Oliveira J.H., Grube A., Kock M., Berlinck R.G., Macedo M.L., Ferreira A.G., Hajdu E. (2004). Ingenamine G and Cyclostellettamines G-I, K, and L from the New Brazilian Species of Marine Sponge *Pachychalina* sp. J. Nat. Prod..

[107] Baldwin J.E., Whitehead R.C. (1992). On the Biosynthesis of Manzamines. Tetrahedron Lett..

[108] Meng Z., Fürstner A. (2020). Total Synthesis Provides Strong Evidence: Xestocyclamine A is the Enantiomer of Ingenamine. J. Am. Chem. Soc..

[109] Meng Z., Spohr S.M., Tobegen S., Fares C., Fürstner A. (2021). A Unified Approach to Polycyclic Alkaloids of the Ingenamine Estate: Total Syntheses of Keramaphidin B, Ingenamine, and Nominal Njaoamine I. J. Am. Chem. Soc..

[110] Liang L., Haltli B., Marchbank D.H., Fischer M., Kirby C.W., Correa H., Clark T.N., Gray C.A., Kerr R.G. (2020). Discovery of an Isothiazolinone-Containing Antitubercular Natural Product Levesquamide. J. Org. Chem..

[111] Jiang Y., Liu X., Li W., Jiao X., Xie P. (2023). Total Synthesis of (−)-Levesquamide. J. Org. Chem..

[112] Hua H.M., Peng J., Dunbar D.C., Schinazi R.F., Andrews A.G.D.C., Cuevas C., Garcia-Fernandez L.F., Kelly M., Hamann M.T. (2007). Batzelladine alkaloids from the caribbean sponge *Monanchora unguifera* and the significant activities against HIV-1 and AIDS opportunistic infectious pathogens. Tetrahedron.

[113] Abd Rani N.Z., Lee Y.K., Ahmad S., Meesala R., Abdullah I. (2022). Fused Tricyclic Guanidine Alkaloids: Insights into Their Structure, Synthesis and Bioactivity. Mar. Drugs.

[114] https://www.newtbdrugs.org/pipeline/clinical.

[115] Lee R.E., Hurdle J.G., Liu J., Bruhn D.F., Matt T., Scherman M.S., Vaddady P.K., Zheng Z., Qi J., Akbergenov R. (2014). Spectinamides: A New Class of Semisynthetic Anti-Tuberculosis Agents that Overcome Native Drug Efflux. Nat. Med..

